# Investigating Changes in pH and Soluble Solids Content of Potato during the Storage by Electronic Nose and Vis/NIR Spectroscopy

**DOI:** 10.3390/foods11244077

**Published:** 2022-12-16

**Authors:** Ali Khorramifar, Vali Rasooli Sharabiani, Hamed Karami, Asma Kisalaei, Jesús Lozano, Robert Rusinek, Marek Gancarz

**Affiliations:** 1 Department of Biosystems Engineering, University of Mohaghegh Ardabili, Ardabil 56199-11367, Iran; 2 Department of Electric Technology, Electronics and Automation, University of Extremadura, Avda. de Elvas S/n, 06006 Badajoz, Spain; 3 Institute of Agrophysics, Polish Academy of Sciences, Doświadczalna 4, 20-290 Lublin, Poland; 4 Faculty of Production and Power Engineering, University of Agriculture in Kraków, Balicka 116B, 30-149 Krakow, Poland

**Keywords:** gas sensor, VOCs, chemometrics, non-destructive methods

## Abstract

Potato is an important agricultural product, ranked as the fourth most common product in the human diet. Potato can be consumed in various forms. As customers expect safe and high-quality products, precise and rapid determination of the quality and composition of potatoes is of crucial significance. The quality of potatoes may alter during the storage period due to various phenomena. Soluble solids content (SSC) and pH are among the quality parameters experiencing alteration during the storage process. This study is thus aimed to assess the variations in SSC and pH during the storage of potatoes using an electronic nose and Vis/NIR spectroscopic techniques with the help of prediction models including partial least squares (PLS), multiple linear regression (MLR), principal component regression (PCR), support vector regression (SVR) and an artificial neural network (ANN). The variations in the SSC and pH are ascending and significant. The results also indicate that the SVR model in the electronic nose has the highest prediction accuracy for the SSC and pH (81, and 92%, respectively). The artificial neural network also managed to predict the SSC and pH at accuracies of 83 and 94%, respectively. SVR method shows the lowest accuracy in Vis/NIR spectroscopy while the PLS model exhibits the best performance in the prediction of the SSC and pH with respective precision of 89 and 93% through the median filter method. The accuracy of the ANN was 85 and 90% in the prediction of the SSC and pH, respectively.

## 1. Introduction

Potato is one of the prominent agricultural products cultivated throughout the world. It is a rich source of carbohydrates, proteins, sugars, and various vitamins. It is native to Peru in South America. After wheat, rice, and corn, potato is the fourth most consumed agricultural product that can be served in various forms (fried, mashed, and chips) [1,2].

Regarding the high expectation of the customers for the safety and quality of the food products, precise, rapid, and targeted determinations of the properties of food products are of crucial significance [3,4]. In the case of potatoes, evaluation of the quality after harvest and sorting is highly important for presenting a reliable and marketable product as the ripening and quality of potatoes are not uniform in the harvesting step [5].

The nutritional and chemical compositions of potatoes vary depending on their cultivar, storage time, nutrition before harvest, and soil type. It is, however, composed of 70–78% water, 16–24% starch, and low amounts of fat, protein, and minerals [6]. Regarding its starch, vitamin, and inorganic salts such as calcium, phosphorous, and iron, the potato has been widely employed to enhance the immune system and cancer prevention [7].

The quality of raw potato is first assessed based on its appearance (size, shape, color, and tuber state); however, the quality of this product can be generally determined by assessing the quality of the final product. Potato is subjected to various phenomena during storage, cooking, or processing that can affect its final quality [8]. Cultivar, physical and chemical compounds, and storage conditions of potato (after harvest) can influence the cooking features and final product of the potato [9].

Several indices have been proposed for the evaluation of the quality of potatoes during their cultivation and storage. Soluble solid content (SSC) is a key index in the quality of the potato as it is directly associated with its nutritional value and taste [10]. Solid substances usually contain sugar, acids, vitamins, and minerals with a critical role in the potato taste [11]. Nevertheless, the SSC distribution on the product differs depending on the growth environment conditions such as temperature, humidity, and light, which may result in nonuniform quality [12].

pH is the other quality index. During industrial processes, proteins of potatoes are often obtained through compounding with acids and heat treatment of water content, which can result in denaturation and irreversible precipitation of protein. Additionally, acidification can also affect the surface activity of proteins [13]. Moreover, pH alteration within the cells (especially due to temperature variation) can signal various processes [14]. On the other hand, this parameter directly affects the rheological features of the starch mud of potatoes. Variations in the pH of potatoes can alter the content of resistant starch, which can subsequently influence its nutritional value [15].

The conventional methods to evaluate the internal quality of potatoes are inefficient due to their low accuracy, time-consuming process, high costs, and sample preparation requirements [9].

Vis-NIR spectroscopic techniques are widely used as alternative potato quality monitoring techniques as they are non-destructive, efficient, rapid, precise, low-cost, and non-contaminating with no need for sample preparation [16]. This technology relies on the absorption of radiation in the Vis-NIR region. NIR spectroscopy has been used for quality control of food and agricultural products [17,18,19], such as citrus fruit and mango [20], tomatoes [21,22] and winter wheat leaf [23], also beverages [24]. An electronic nose (E-nose) can also play a decisive role in the quality determination of the agricultural product [25]. This device employs a different approach to classify and determine the quality of the products [26,27], through detection of odor and volatile compounds [28,29].

Similar to pH measurement, conventional methods of SSC measurement are often destructive and time-consuming. E-nose and spectroscopic methods are capable of assessing the quality features of food products in a fast and non-destructive manner [9]. Multivariable statistical analysis methods can also be utilized for the prediction and determination of chemical compounds within food samples [30].

Some studies have been conducted to test the near-infrared spectroscopy to measure quality parameters of potatoes, such as sugars or dry matter content [31,32,33,34]. Escuredo et al. [35] estimated potato quality parameters using a portable near-infrared spectrometer. They used principal component analysis and modified partial least squares regression method to develop the NIR calibration model. The best determination coefficients obtained for dry matter and reducing sugars were of 0.72 and 0.55, respectively. NIR technology has been extensively studied for homogenized samples of potatoes, such as potato pulp, sliced potatoes, freeze-dried potato, and cooked potato mash [36], but to a lesser extent in intact potato [32].

However, to the best of authors’ knowledge, no information has been published on the application of an electronic nose in predicting the pH and SSC value in potatoes. Yu and Wang [37] employed an E-nose in combination with an ANN and Linear discriminant analysis (LDA) to determine and differentiate the quality of green tea leaves, and they managed to detect the quality of this product with an accuracy of 100% using both methods. Qiu and Wang [38] applied E-nose and E-tongue along with the LDA model to determine the quality of citrus. They showed that the LDA model can detect the quality of citrus with accuracies of 95.8% and 97.6% in combination with E-nose and E-tongue, respectively. Zhou et al. [39] applied E-nose and PCR and MLR methods for predicting the linalool content of Osmanthus fragrans with respective accuracies of 99.2 and 89.5%. Govari et al. [40] utilized the E-nose and PLSR model for rapid evaluation of the microbiological quality of Abramis brama orientalis fillet. Their results indicated that the accuracy of the spectroscopic method was far higher than the E-nose, and the E-nose showed the lowest precision.

This research aimed to investigate and compare the ability of E-nose and Vis/NIR spectroscopy using chemometrics and artificial neural network methods to reliably detect the pH and SSC changes of potato.

## 2. Materials and Methods

### 2.1. Sample Preparation

In this research, 8 kg potato (Sante cultivar) was provided from the agricultural research center of Ardabil city immediately after their harvest. The samples were tested and grouped into four periods (once immediately after harvest and three times during their storage in two-week intervals). The tests were carried out considering 15 replications. The acquired data included E-nose data, Vis/NIR spectral data, SSC, and pH.

### 2.2. Data Acquisition by E-Nose

**Figure 1:**
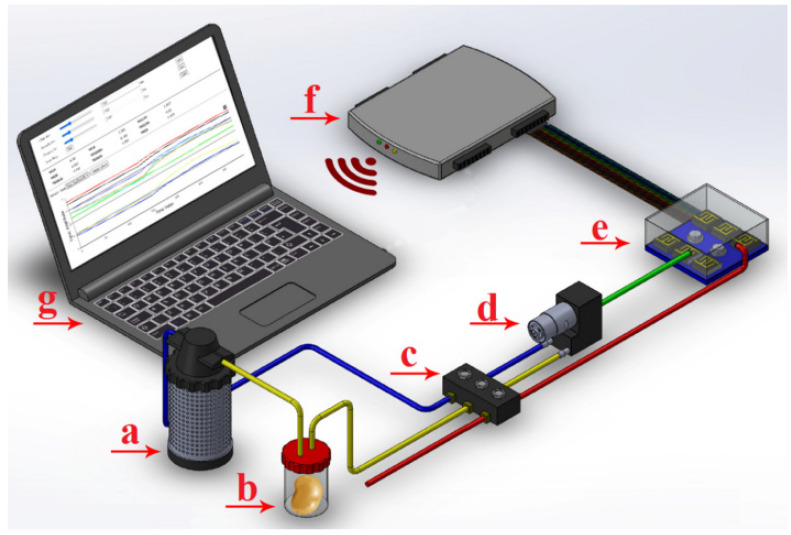
The schematic of an electronic nose and its components a—air filter (activated charcoal for removing ambient-air volatile organic compound (VOC) hydrocarbons), b—headspace chamber of the sample, c—solenoid air valves, d—pump (Diaphragm type), e—sensor array chamber, f—data acquisition recorder and wireless transfer card, and g—laptop [1].

The applied E-nose included nine metal oxide semi-conductor (MOS) sensors (the most common commercial sensor to detect volatile gas (Figure 1) [41]. The mentioned sensor was as follows: (1) MQ9 (for CO and combustible gases), (2) MQ4 (for urban gases and methane), (3) MQ135 (for benzene, ammonia sulfide), (4) MQ8 (for hydrogen), (5) TGS2620 (for alcohols, organic solvents), (6) MQ136 (for sulfur dioxide), (7) TGS813 (for CH4, C3H8, and C4H10), (8) TGS822 (for organic solvents), and (9) MQ3 (for alcohols). In a typical data acquisition process, several potatoes were placed in a plastic chamber for 6 h until the chamber was saturated with the odor of the samples [42,43]. Sensors showed voltage variations relative to the emitted smell of the samples and their output responses were recorded in one-second intervals. After data acquisition, the baseline was corrected with the help of Equation (1) to eliminate any noise and possible deviations. The following equation was also used for obtaining a normalized and dimensionless sensor output [44]:

(1) $$Y_{s}(t)=\frac{X_{s}(t)-X_{s}(0)}{X_{s}(0)}$$

in which, *Y_s_*(*t*), *X_s_*(0), and *X_s_*(*t*) are the normalized response, baseline, and sensor response, respectively.

### 2.3. Data Acquisition by Vis/NIR Spectroscopy

**Figure 2:**
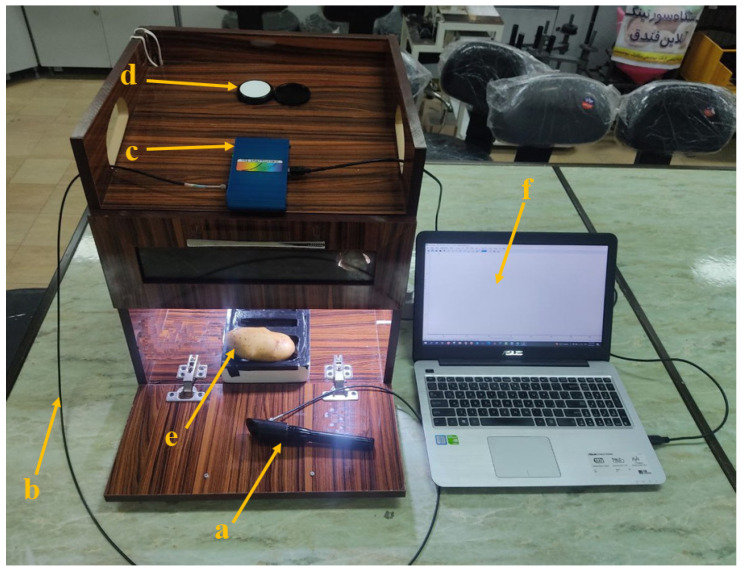
Spectroscopic device a—reflectance probe, b—fiber-optic cable, c—PS-100, d—reflectance standard, e—sample, and f—laptop.

Vis/NIR spectroscopic tests were carried out by a spectroradiometer (Model PS-100; Apogee Instruments, INC., Logan, UT, USA) equipped with a 2048-pixel CCD detector with a resolution of 1 nm and halogen tungsten light source in the wavelength range from 350 to 1100 nm. A standard disc was employed to calibrate the device (Figure 2).

#### 2.3.1. Data Acquisition Process

Before NIR-based quality assessments, potatoes (and other agricultural products) should be cleaned to reduce signals caused by surface impurities (e.g., soil) [45]. Impurities on the potato surface (such as soil) have lower absorption and higher reflection, while the inner parts of the potato have higher absorption due to their high moisture and dry matter [46].

For each sample, spectrometry was performed by Spectra-Wiz Spectrometer OS v5.33 (c) 2014 software, and the data were recorded after averaging. This software directly extracts absorption data with no need for conversion.

After saving the spectra and their transfer to Excel software, the spectra of each sample were averaged and recorded. The initial and final wavelengths of the obtained spectra were removed due to the presence of noise; finally, the spectral range from 600 to 950 nm was considered [47].

#### 2.3.2. Preprocessing of Spectral Data

Spectral data are under the influence of various factors such as light scattering, the surface roughness of the sample, the size of the samples, and noise (due to the increased temperature of the spectrometer). This unwanted information can affect useful information, decreasing the accuracy of calibration models. To reduce the effects of undesired information, stable, reliable, and accurate calibration models are necessary to pre-process spectral data. Various preprocessing methods have been developed each for a specific purpose. The choice of the right pre-processing method is based on trial and error and it is not possible to use a specific pre-processing method for all the prediction models [48].

In this research, various smoothing pre-processing methods (Savitzky–Golay (SG), Gaussian, median, and moving average) were employed. Smoothing filters (e.g., Gaussian, median, moving average, and SG filters) can be applied to reduce the noise of the spectral data. It should be noted that although these filters can significantly reduce noise, they must be used with care to avoid altering important data. Although smoothing improves the condition of the Vis/NIR spectrum, it may cause the loss of useful information. Despite numerous studies on the selection of optimal points, this selection is mostly conducted experimentally. An empirical rule states that the width of the number of optimal points for spectrum smoothing should not be greater than the width necessary to cover half of the smallest peak in the spectrum [49].

### 2.4. Sugar Content Measurement

The sugar content of the samples was measured in each period using a liquid refractometer (HI96801; HANNA instruments company, Woonsocket, RI, USA). In a typical process, the water of the samples was removed and placed through a microtube inside the refrigerated centrifuge (high-rate) LISA France model. The impurities were settled after rotating at 1800 rpm for 120 s; the potato juice was placed on the refractometer after reaching the ambient temperature and its sugar content was read according to the Brix index [1].

### 2.5. pH Measurement

**Figure 3:**
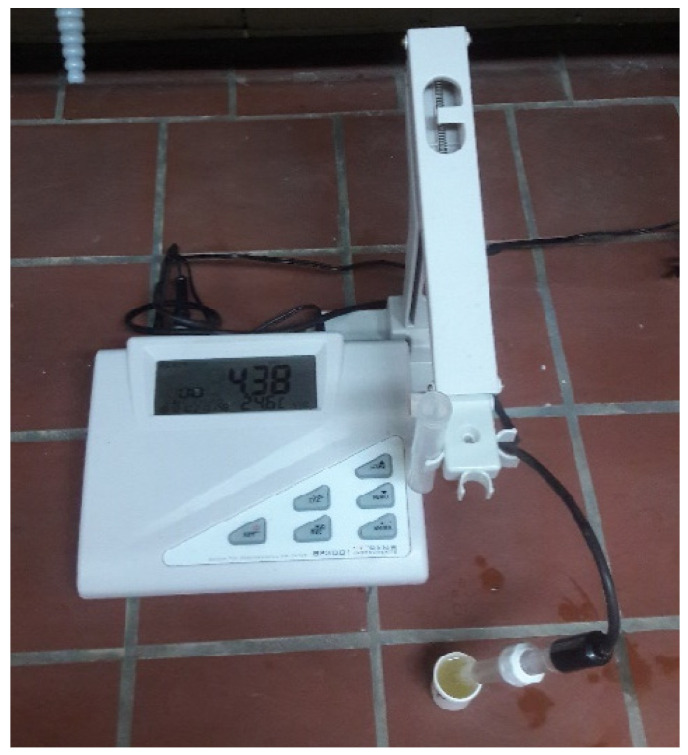
BP 300 digital pH meter.

A BP 300 digital pH meter was utilized to measure the pH of potato samples (Figure 3). After each measurement, the electrode of the pH meter was washed with distilled water and dried with tissue to prevent errors in the next measurements.

### 2.6. Data Modeling

Chemometrics uses multivariate statistics to obtain useful information from complex analytical data. In this study, multivariate calibration models were produced with the help of multiple linear regression (MLR), principal component regression (PCR), and partial least square regression (PLSR) models to explain the model and relationships between the E-nose and spectroscopic data with the chemical properties (sugar and acidity).

MLR establishes a linear relationship between a dependent variable (y) and a set of several explanatory variables (x). This model can be employed in cases where the number of variables is less than the number of samples for weakly related variables [50].

PCR reduces the number of explanatory variables by selecting a few principal components (PCs) instead of the original ones. This method may be applied in two stages. In the first stage, it provides the possibility of determining the main components by the PCA method and allows to obtain an unrelated matrix of variables. The second stage involves the development of the MLR model using the principal components as variables. The calibration model does not have enough information to make a correct prediction when the principal components are too small. In cases with too many main factors, unwanted information such as experimental errors or noise will be introduced to the model [9].

PLSR, also known as PLS, is a new method of multivariate statistical analysis commonly used for developing multidimensional calibration models. PLSR can process linear data and reduce the number of calibration samples required, making it a gold standard in chemometric analyses [3]. PLS is a stationary linear regression technique (of Y = AX + B type) that reduces the size of variables by extracting linear combinations from the original sample (X). These combinations are called (A) orthogonal latent components. It is important to consider a set of validation data as a supervised technique to select the optimal number of latent variables [3]. The PLS method can be more effectively used when the dependent variables have a higher linear correlation. This is a bilinear model based on the matrices of *X* (independent variables) and *Y* (dependent variable), which can be considered as external and internal relations, respectively.

Proposed by Drucker et al. [51], support vector regression (SVR) is an extension of the support vector machine (SVM). SVR is a machine learning method that can be employed to study non-parametric estimation problems in limited-sample situations, making it suitable for small samples and non-linear problems [52].

In this research, the output data of the E-nose and spectrometer were determined as the independent variable (x), while the measured data of sugar and acidity were considered as the dependent variable (y) and entered into the Unscrambler software (version X 10.4, CAMO, Trondheim, Norway).

An artificial neural network was also used to predict the SSC and acidity of the potato samples using MATLAB R2013a software. The hidden layer with the optimal number of neurons were considered by trial and error, and 60 and 350 input neurons were taken for the E-nose and NIR spectrometer, respectively (according to the number of output data). The network was trained by the logarithmic sigmoid transfer function through the Levenberg–Marquardt method. For learning, testing, and validation, 70, 15, and 15% of the data were randomly selected. Training data were fed to the network during training and the network was adjusted according to their errors. Validation was also employed to measure network generalization and training completion. Data testing had no impact on the training and provided an independent measure of network performance during and after the training process [53]. It is worth mention that the data must first be normalized to be used in the ANN method for modeling and prediction purposes, for which Equation (2) was used.

(2) $$X=\frac{Xi-X\min}{X\max-X\min}$$

### 2.7. Statistical Analysis

The SSC and pH levels in potato were determined according to a completely randomized experimental design with four treatments (four different storage times: every two weeks) and fourteen replicates per period, resulting in 4 × 14 = 56 total replicates per treatment (n = 56). The statistical data analysis was performed using Minitab 16 software (Minitab, LLC., State College, PA, USA).

## 3. Results and Discussion

### 3.1. Variance Analysis of SSC and pH

The SSC and pH levels of the potato samples were measured by a refractometer and pH meter, respectively. The corresponding ANOVA analysis results are presented in Table 1.

The ANOVA results of the SSC and pH were significant at levels of 5% and 1%, with the corresponding coefficient (CV) of variation of 6.77 and 7.18, respectively.

**Figure 4:**
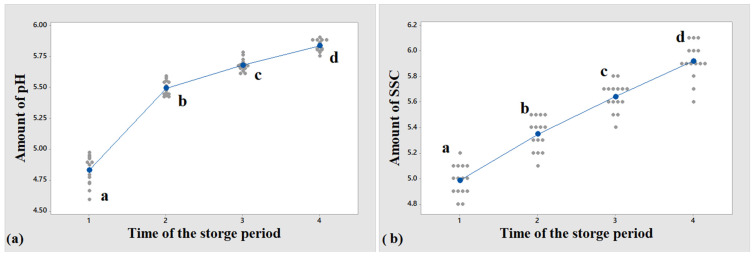
Mean variations in (**a**) pH and (**b**) SSC of the potatoes during the storage process.

The average SSC (in terms of Brix index) and pH values are compared and presented in Table 2 and Figure 4, respectively. According to the SSC results, the post-harvest SSC of the potato sample increased over time after harvesting due to the hydrolysis of starch (the main compound in potato tubers) as a result of the respiration of the product, which turned starch into sugar [54]. In addition to the starch decomposition, the loss of product moisture can also increase the potato SSC [55]. A study on potato compounds stated a decline in the sugar content of potatoes during the storage period [56]. This discrepancy can be assigned to the difference in the cultivars as well as the harvest time of the product. Regarding acidity, the pH variations rose during the storage period, such that the pH value increased slightly during the storage period. At the time of harvesting, the pH of potatoes was somewhat lower than the storage period. These results were consistent with the findings of Paik [57]. The pH values were also consistent with the reports of José Carlos Feltran et al. [58] who measured the pH of 20 different potato cultivars and reported no significant difference in the pH of different potato cultivars.

In the above table, T1 is associated with the harvesting step; while T2–T4 are related to the storage period (with two-week intervals).

### 3.2. E-Nose Findings

**Figure 5:**
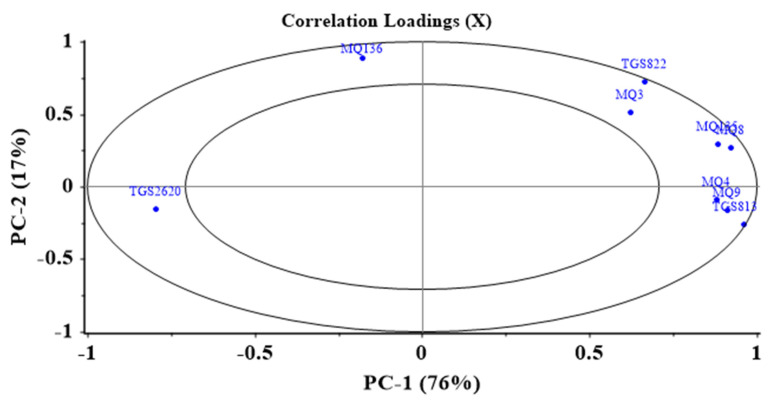
Loading diagram showing the effectiveness of individual MOS sensors in detection of potato odor.

A correlation loading plot was employed to determine the ability of the sensors to detect the potato odor. In these diagrams, the higher the loading coefficient of the sensors, the higher their contribution to the detection of the sample odor. On the other hand, the lower the loading coefficient (the closer to the middle circle), the lower its role and influence on the results [1]. Therefore, MQ135, TGS813, and TGS822 had the most important roles in data classification (Figure 5).

Identifying the most prominent sensors in odor detection, these sensors can be employed to develop the most effective and efficient E-nose to simultaneously reduce the response time of the E-nose and the complexity of the analysis (lower pre-training by extra data) [59].

**Figure 6:**
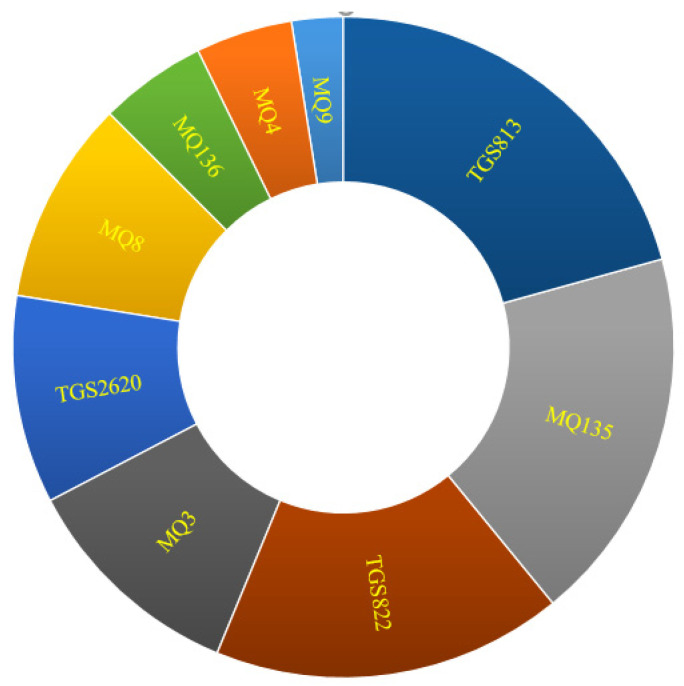
Sunburst diagram showing the role of sensors in odor detection.

The Sunburst diagram (Figure 6) also depicts the role and sensitivity of the sensors to the potato odor. As seen, TGS813, MQ135, and TGS822 had the highest efficiency, respectively, while MQ9 exhibited the lowest impact.

An E-nose was employed as a non-destructive method to predict the SSC content and pH of the potato samples using various statistical methods such as PCR, MLR, PLS, and SVR, whose results are listed in Table 3.

According to Table 3, the E-nose can predict the pH values with suitable accuracy (R^2^ = 83% for PCR, MLR, and PLS models and 92% accuracy for the SVR model). However, regarding the SSC prediction, the PCR, MLR, and PLS models exhibited low accuracy (R^2^ = 0.64–0.66), while the SVR model managed to predict the SSC of potatoes with high precision (93%). The results of this research (about acidity) were consistent with the reports by Huang and Gu [60], who used a sensor array and machine learning (SVR) to distinguish pork-fake beef with an accuracy of 92%. In another research, Wu et al. [61] utilized an E-nose to detect and predict the contamination of sweet potato with C. Fimbriata with a respective accuracy of 65 and 66% for PLS and PCR, which is very close to the results of this research on the SSC.

According to Table 3, the accuracies of the PCR, MLR, and PLS models were generally very close to each other (for both SSC and pH) and the accuracy of the SVR model was higher than all models. Therefore, among the tested models, SVR is the best model for predicting the SSC and pH values in potatoes using an E-nose.

### 3.3. Artificial Neural Network Results

**Figure 7:**
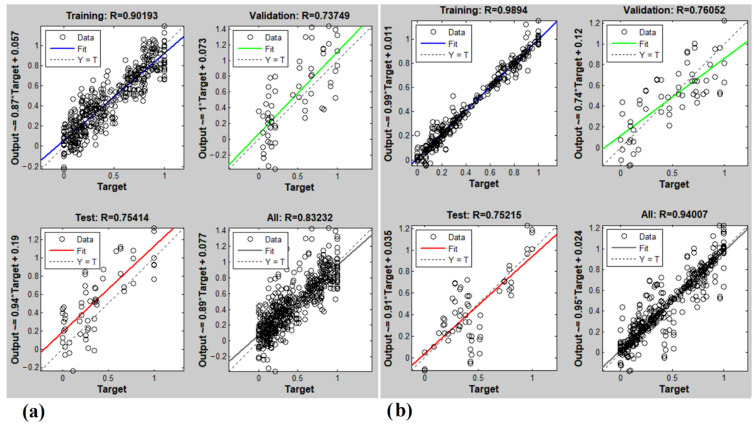
(**a**) SSC and (**b**) pH predicted by ANN using an E-nose.

Figure 7 shows the ANN-predicted SSC content and pH of potatoes using an E-nose. Accordingly, the correlation between the observed values of the response variable and the predicted values of the response variable made was 83 and 94% for the SSC and pH, respectively.

The results of this research on predicting the SSC content are in line with the findings of a study addressing the prediction of oxidation in edible oil using an E-nose and ANN [44]. The prediction accuracy for the SSC was also very close to that of Yu et al. [62] who managed to classify green tea with an accuracy of 85% using an ANN and E-nose.

### 3.4. Vis/NIR Spectroscopic Results

Smoothing filters such as Gaussian, moving average, and SG were applied to decline the noise in the acquired spectral data and improve the quality of the Vis/NIR spectrum. The influence of any of these smoothing methods on the accuracy of the prediction models can be found in Table 4.

The MLR model cannot be used for our spectral analysis, because as mentioned in the Materials and Methods section, this model can be employed in cases where the number of samples is more than the number of variables, which is the opposite in the case of spectroscopic data (unlike the E-nose where the number of samples is greater than the number of variables). On the other hand, the models with $R_{val}^{2}\leq 0.6$ can be eliminated [63], as the response of the spectra to the SSC and pH of potato does not have a direct or acceptable relationship. Therefore, the SVR model is not sufficiently accurate in predicting the SSC and pH values of the samples, and it is considered an ineffective model in the Vis-NIR spectroscopic method, while this model offered higher precision in the E-nose method. According to the table, the R^2^ of the PLS model is the highest in all cases and it more reliably predicts the SSC content and especially the pH level.

Concerning the prediction of the SSC and pH by spectrometry (Table 4), PLS is the best model as it offers the highest R^2^ in the median filter smoothing method with R^2^ = 0.801 and RMSE = 0.168 for the SSC and R^2^ = 0.931 and RMSE = 0.104 for the pH.

The accuracy of the results of the current research on pH prediction with the PLSR method was much higher than the results of the research by de Brito et al. [64], who employed [51] Vis-NIR spectroscopy and the PLSR method to predict the pH of tomatoes with R^2^ = 0.59.

NIR spectroscopy was also utilized to control the quality of cashew apple and guava nectar, in terms of quality parameters such as total pH. The results showed that conventional methods can be replaced by NIR leading to rapid, easy, and safe processing of many parameters [65].

Farhadi et al. [66] employed Vis-NIR spectroscopy to detect potato contents (starch, reducing sugar, and moisture) and managed to determine these contents at high accuracy using the PLS model (92–98%).

**Figure 8:**
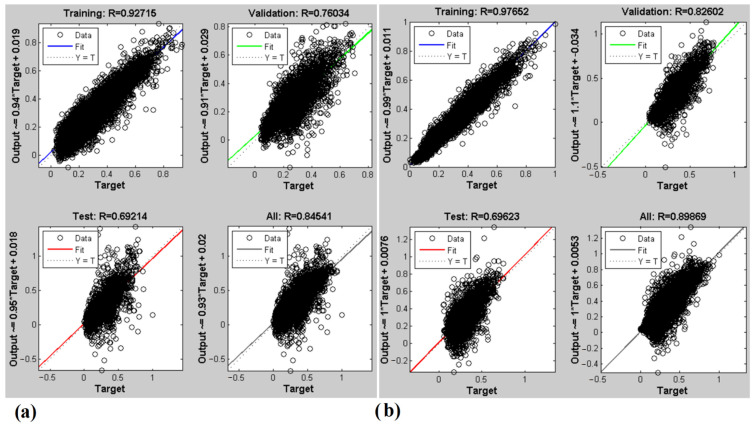
ANN results with NIR spectroscopy to predict (**a**) SSC and (**b**) pH.

Figure 8 presents the ANN-predicted SSC and pH for the spectrometry method. As shown, the ability of the ANN to detect the SSC is the same for both E-nose and NIR spectroscopy methods (R^2^ = 0.84). Concerning the pH prediction, the ANN with spectrometry offered a higher accuracy (90%) compared to the E-nose.

## 4. Conclusions

This research was carried out to develop a suitable model for reliable prediction of potatoes’ SSC and pH values during the storage period using E-nose and Vis/NIR spectroscopy. According to the results, the SSC and pH values can be detected during the storage period using the E-nose and the SVR model with respective R^2^ of 81 and 92%. Using the ANN, there will be a 2% enhancement in the prediction performance of the SSC and pH (R^2^ = 83 and 94%). Vis/NIR spectroscopy was also employed to detect the mentioned parameters during the storage period. After applying different smoothing methods (to reduce the noise), it was found that the Vis/NIR spectroscopy method, combined with the PLS model and median filter smoothing method, can predict the SSC and pH values at the highest accuracies (80 and 93%, respectively). The accuracy of the ANN method was also 85 and 90%, respectively. Accordingly, an electronic nose combined with the ANN provided the most promising performance in the prediction of the SSC and pH. The findings of this research can be employed in diverse food industries, including the production of potato chips and mashed potatoes to offer a final product with the best quality to the market.

## Figures and Tables

**Table 1:** Analysis of variance of chemical parameters of potato.

Sources	Degrees of Freedom	Mean of Squares
SSC	3	2.40089 **
Error	56	0.01598
Total	59	
pH	3	2.91903 **
Error	56	0.00527
Total	59	

** significant at *p* ≤ 0.01.

**Table 2:** Result of Tukey mean comparison test for SSC and acidity of potato (α = 0.05).

	T1	T2	T3	T4
SSC	4.9867 ^a^	5.3467 ^b^	5.6400 ^c^	5.9200 ^d^
Acidity	4.8300 ^a^	5.4900 ^b^	5.6760 ^c^	5.8333 ^d^

The letters ^a^, ^b^, ^c^, and ^d^ describe significant differences between the mean values.

**Table 3:** E-nose-determined pH and SSC content of potato samples predicted by various models.

Variable	Model	R^2^val	R^2^cal	RMSEval	RMSEcal
pH	PCR	0.830	0.877	0.162	0.136
	MLR	0.823	0.877	0.165	0.149
	PLS	0.829	0.865	0.164	0.085
	SVR	0.923	0.958	0.112	0.345
SSC	PCR	0.655	0.748	0.219	0.184
	MLR	0.638	0.748	0.223	0.202
	PLS	0.664	0.747	0.217	0.185
	SVR	0.807	0.877	0.165	0.134

**Table 4:** SSC and acidity of the potato samples measured by NIR spectroscopy and predicted by various models.

Variable	Smoothing	Model	R^2^val	R^2^cal	RMSEval	RMSEcal
SSC	Moving Average	PCR	0.716	0.825	0.199	0.153
		PLS	0.770	0.938	0.179	0.092
		SVR	0.551	0.701	0.253	0.215
	Gaussian Filter	PCR	0.781	0.878	0.174	0.128
		PLS	0.789	0.943	0.171	0.087
		SVR	0.540	0.687	0.255	0.219
	Median Filter	PCR	0.711	0.823	0.201	0.155
		PLS	0.801	0.943	0.168	0.088
		SVR	0.526	0.668	0.259	0.224
	Savitzky–Golay	PCR	0.746	0.869	0.189	0.133
		PLS	0.787	0.937	0.173	0.093
		SVR	0.515	0.653	0.261	0.228
pH	Moving Average	PCR	0.871	0.927	0.141	0.105
		PLS	0.906	0.971	0.121	0.066
		SVR	0.496	0.770	0.308	0.252
	Gaussian Filter	PCR	0.872	0.929	0.141	0.103
		PLS	0.914	0.978	0.116	0.058
		SVR	0.569	0.806	0.292	0.232
	Median Filter	PCR	0.684	0.803	0.210	0.163
		PLS	0.931	0.984	0.104	0.049
		SVR	0.523	0.789	0.302	0.243
	Savitzky–Golay	PCR	0.872	0.929	0.142	0.104
		PLS	0.920	0.981	0.111	0.053
		SVR	0.498	0.770	0.308	0.252

## Data Availability

The datasets used and/or analyzed during the current study are available from the corresponding author on reasonable request.
